# Microwave Hydrodiffusion and Gravity Extraction of Vitamin C and Antioxidant Compounds from Rosehips (*Rosa canina* L.)

**DOI:** 10.3390/foods12163051

**Published:** 2023-08-15

**Authors:** Eugenia Mazzara, Giovanni Caprioli, Gianmarco Simonelli, Ahmed M. Mustafa, Filippo Maggi, Marco Cespi

**Affiliations:** 1 Chemistry Interdisciplinary Project Research Center, School of Pharmacy, University of Camerino, Via Madonna delle Carceri 9/B, 62032 Camerino, Italy; eugenia.mazzara@unicam.it (E.M.); giovanni.caprioli@unicam.it (G.C.); gianmarco.simonelli@studenti.unicam.it (G.S.); ahmed.mustafa@unicam.it (A.M.M.); marco.cespi@unicam.it (M.C.); 2 Department of Pharmacognosy, Faculty of Pharmacy, Zagazig University, Zagazig 44519, Egypt

**Keywords:** rosehips, ascorbic acid, HPLC, antioxidants

## Abstract

Rosehips, *Rosa canina* L. (Rosaceae family), have been used for a long time for their beneficial effects on health, and they are largely exploited in the food and nutraceutical supplement sectors. The aim of this work was to apply and optimize for the first time the microwave hydrodiffusion and gravity (MHG) extraction of rosehips, as a novel application of solvent-free microwave extraction, previously conducted in a similar way only on mulberry, strawberry, and onion. The optimization was performed through a central composite design (CCD) by evaluating the effect of the experimental parameters on the yield; total polyphenol, flavonoid, and anthocyanin contents; radical scavenging activity; and content of vitamin C in the obtained extracts. As a result, the water moistening pretreatment was clearly revealed to possess a pivotal role in the quality of the rosehip extracts. Among the evaluated responses, the yield, the anthocyanin content, and the antioxidant activity were well described by the statistical model. Notably, the optimized MHG extract was compared with the ones obtained by conventional solvent extraction showing higher amounts of vitamin C, polyphenols, flavonoids, and anthocyanins, while the traditional extracts performed better in terms of yield. In conclusion, MHG represents a promising alternative to standard extraction methods for obtaining rosehip extracts rich in vitamin C and antioxidant compounds. In this respect, the results of our research support the employment of MHG on an industrial level for the production of rosehip-based food supplements enriched in vitamin C.

## 1. Introduction

*Rosa canina* L. (Rosaceae), rosehip or dog rose, is an erect shrub up to 3.5 m high, naturally spread over a vast area including the Caucasus, Central and Western Asia, Europe, Northwestern Africa, the northern and western areas of Iran and Iraq, Pakistan, Northern Afghanistan, Russia, Armenia, and Ukraine [1]. This plant grows between 30 and 1700 m a.s.l. mostly on alkaline soils [2]. It is easily adaptable to extreme climate conditions and can develop on sandy, loamy, argillaceous, rocky, humid-dry, and shallow to deep soils [3]. *R. canina* false fruits, rosehips, have been employed to produce jams, marmalades, wine, juices, teas, puddings, bakery products, extruded foods [1], Swedish commercial soups, and also brandy in Hungary [4]. Some traditional uses of rosehips encompass the prevention of cold and other infections, diuretic effects, and the treatment of several inflammatory conditions, especially arthritis, rheumatism, gout, and sciatica [5]. Rosehips are reported to contain numerous compounds that are responsible for their positive health benefits, including polyphenols, flavonoids, carotenoids, and especially vitamin C, whose average content has been reported to be 550 mg/100 g of rosehip flesh [6]. Besides the traditional extraction methods employed for rosehips, such as Soxhlet with organic solvents like *n*-hexane [7] or water infusion [8], new eco-friendly processes, aimed at obtaining high quality rosehip oils and extracts, are emerging. Among them, ultrasound-assisted extraction by deep eutectic solvents [9] has been optimized through response surface methodology to evaluate the phenolic compound contents and antioxidant activity in *R. canina* extract. Moreover, supercritical CO_2_ extraction optimization of rosehip oil recovery led to a decrease in the peroxide number as a sign of oxidative degradation [10] and to enhanced content of essential fatty acids and natural antioxidants, namely tocopherols and carotenoids [11]. Additionally, a pulsed electric field was applied to improve the polyphenolic profile of *R. canina* aqueous extract [12]. The technique of microwave-assisted extraction (MAE) has been employed on rosehips to obtain a valuable oil at a high yield [7] and extracts rich in antioxidant compounds [8]. Microwave-assisted hydrodistillation has been performed on *R. canina* roots to produce an essential oil with an increased quality and number of volatile constituents [13].

Notably, no literature reports can be found about microwave hydrodiffusion and gravity (MHG) extraction of rosehips. During this process, fresh plant material is subjected to microwaves, in situ moisture of the biomass is heated, and cells are damaged releasing their bioactive molecules that are drained in favor of gravity, passing through a condenser located below the reactor. In this way, a fresh raw juice (aqueous extract) is continuously produced and recovered. This solvent-free method can be regarded as one of the most promising candidates for industrial scaleup of extraction procedures, especially in the agrifood industry. A considerable saving of time, energy, and plant material has been proven for MHG, with respect to conventional extraction tools [14]. In particular, this system works in favor of gravity, without requiring additional energy consumption that is needed when employing other extraction methods which act against gravity. In previous reports, MHG has been successfully applied to obtain mulberry [15], strawberry [16], and onion [17] extracts.

In this framework, our objective was to evaluate the feasibility of the MHG process for rosehip extraction. For the purpose we studied the influence of several experimental parameters on the qualitative–quantitative features of the produced extracts. This research has been conducted through a design of experiment (DoE) statistical approach, and the results were compared with those obtained by the conventional maceration with water or hydroalcoholic mixtures.

## 2. Materials and Methods

### 2.1. Plant Material

The rosehips of *R. canina* were collected in Torre Beregna, Camerino (700 m a.s.l., 43°10′16.6″ N; 13°06′42.0″ E) in November 2020 and were then immediately frozen and stored. A plant herbarium specimen was identified by one of us (F.M.) and archived in the Herbarium Camerinensis c/o School of Bioscience and Veterinary Medicine, University of Camerino, under the codex CAME#29334. The water content of rosehips was 55.4 ± 0.7%, determined using a thermobalance (Scaltec SMO 01, Goettingen, Germany) operating at 100 °C on 3 randomly collected samples of around 1 g.

### 2.2. Sample Extraction

#### 2.2.1. Microwave Hydrodiffusion and Gravity (MHG) Extraction

The rosehip juice was extracted through an advanced microwave extraction system (ETHOS X, Milestone, Milan, Italy), consisting of a microwave reactor of 2.45 GHz, two magnetrons with a maximum delivering power of 1800 W (2 × 950 W) and an infrared sensor to monitor the temperature. All the extractions were conducted at atmospheric pressure using a 5 L glass reactor (Pyrex, Charleroi, PA, USA) closed with a glass cover. The MHG system was configured using the “Flavors’ setup” coupled with a Chiller (Smart H150-2100S, Labtech S.r.l., Sorisole, Italy), to keep the water temperature at 8 °C.

All the extractions were performed on 200 g of rosehips preliminary shredded on a lab scale grinder, setting the MHG experimental conditions, namely the microwave irradiation power (MP, W/g), extraction time (ET, min), and water moistening pretreatment (MO, yes or no), as reported in Section 2.3.1. The temperature inside the reactor did not exceed 80 °C during all the extraction runs. The water moistening pretreatment was carried out by immersing under magnetic stirring 200 g of shredded rosehips in 200 mL of boiling water for 15 min prior to the MHG extraction. The juice or crude extract that fell under the effect of gravity was collected in a graduated baker positioned below the instrument. The juice was then filtered and freeze-dried. The dry extracts (DE) were stored at −18 °C until analysis.

#### 2.2.2. Conventional Solvent Extraction

Fifty g of shredded rosehips were extracted using 250 mL of distilled water or a 1:1 water/ethanol mixture. The process was carried out under magnetic stirring (M2-A, Argo Lab, Carpi, Italy) at ambient temperature for 1 h. The extraction liquid was filtered and then lyophilized. The obtained powders were stored at −18 °C before analysis. For the extraction performed using the hydroalcoholic mixture, ethanol was removed prior to the lyophilization using a rotavapor. For each solvent, the extraction was performed in triplicate.

### 2.3. Design of Experiment (DoE)

#### 2.3.1. Central Composite Design (CCD)

The understanding and modeling of the effect of the experimental parameters on qualitative and quantitative features of rosehip juice extracted by MHG were carried out through the application of a response surface methodology (RSM), the central composite design (CCD). The experimental parameters investigated were the microwave irradiation power (MP, W/g) and extraction time (ET, min), two numerical factors, and the water moistening pretreatment (MO, yes or no) which represents a categorical (binary in this case) factor. The CCD for 2 numerical and 1 categorical factor was composed of 16 runs (8 factorial points, coded 1 or −1; 8 axial points coded 1.41/0 or −1.41/0) with the addition of 2 replicates of the central point for each level of the binary factor (coded as 0). The absolute values of the level 1 and −1 of the factors MP and EP were chosen according to our experience of MHG [15], and more in general of the MAE process [18,19,20], while the choice of including the factor MO was due to the results reported in the literature on antioxidants and vitamin C content of rosehips and broccoli extracts [21,22]. The entire list of all the experimental runs is shown in Table 1. 

The juice extracted from each MHG run of Table 1 has been characterized in terms of:

- Extraction yield % (Yld), calculated as the weight of lyophilized extract per 100 g of fresh-weight fruit;
- Total phenolic content (TPC), assessed as described in Section 2.4.1;
- Total flavonoid content (TFC), assessed as reported in Section 2.4.2;
- Total anthocyanin content (TAC), assessed as described in Section 2.4.3;
- Radical scavenging activity (DPPH), assessed as reported in Section 2.4.4;
- Vitamin C content (Vit. C), determined as described in Section 2.4.5.

The relationship between each single response (the juice features reported in the points A–F) and the experimental variables MP, ET, and MO was assessed through multilinear regression using a full quadratic model:

(1) $$y=\beta_{0}+\sum_{i=1}^{n}\;\beta_{i}\cdot x_{i}+\sum_{i=1}^{n}\;\beta_{ii}\cdot x_{i}^{2}+\sum_{i<j}^{}\;\beta_{ij}\cdot x_{i}x_{j}$$

where y represents the response, β_0_ is the model constant, β_i_ represents the coefficients of the variables x_i_ (linear terms), β_ii_ represents the coefficients of the variables x_i_^2^ (quadratic terms), and β_ij_ represents the coefficients of the variables x_i_x_j_ (first-order interaction terms). The determined full quadratic models were improved in terms of precision of the estimated coefficients and mean square error by reducing the numbers of terms using a stepwise regression operating in backward elimination mode. This approach generates a set of simplified models, and the most suitable ones were selected through comparison of Mallows’ Cp statistic, the predicted coefficient of multiple determination (R^2^_pred_), and the adjusted coefficient of multiple determination (R^2^_adj_) [23]. The best models determined after the model reduction procedure were assessed using ANOVA, coefficient, and residual analysis. The planning of the DoE and the analysis of all the data were performed with the software Minitab 18. 

### 2.4. Dry Extract Analysis

#### 2.4.1. Total Phenolic Content (TPC)

The TPC was assessed through the Folin–Ciocalteu method, slightly modified with respect to that reported by Mustafa et al. [24,25]. Briefly, dry extracts were dissolved in distilled water (2 mg/mL), and 0.5 mL of rosehip extract solution was allowed to react with 2.5 mL of Folin–Ciocalteu reagent solution (10-fold diluted in water), and 7.5 mL of 7.5% Na_2_CO_3_ aqueous solution. The reaction mixture, after being maintained at room temperature in the dark for 2 h, was analyzed through a Cary 8454 UV–Vis spectrophotometer (Agilent Technologies, Woburn, MA, USA). The absorbance was registered at 765 nm. The TPC quantification was conducted by using gallic acid standard to build a calibration curve. The TPC was indicated as mg of gallic acid equivalent (GAE) per gram (g) of dry extract (DE). The average of two experiments was calculated to express the results. Although the spectrophotometric method is versatile and common, it is non-specific and may overestimate the polyphenolic content, mainly because of non-phenolic materials present in the extracts interfering in the spectrophotometric analysis.

#### 2.4.2. Total Flavonoid Content (TFC)

The TFC determination was carried out spectrophotometrically, based on a previous work [26]. In this case, 0.5 mL of extract solution (prepared as described in Section 2.4.1), 0.15 mL of NaNO_2_ (0.5 M), 3.2 mL of methanol (30% *v*/*v*), and 0.15 mL of AlCl_3_·6H_2_O (0.3 M) were mixed. After waiting for 5 min, 1 mL of NaOH (1 M) was added. After shaking and leaving the mixture at room temperature in the dark for 30 min, the absorbance was read at 506 nm against the blank reagent. The calibration curve for the TFC assay was established by using rutin as a reference compound. The TFC was measured as mg of rutin equivalent (RE)/g DE.

#### 2.4.3. Total Anthocyanin Content (TAC)

The TAC was determined through the pH differential method, previously reported by Avalos-Llano et al. [27]. The extracts were diluted 1:10 into the buffers of 0.025 mol/L potassium chloride at pH = 1 and 0.4 mol/L sodium acetate at pH = 4.5. After 15 min, the absorbance of the extracts diluted at pH = 1 and pH = 4.5 was registered at 510 and 700 nm, respectively. The following formula was used to quantify the TAC:

TAC = [(A_510 nm_ − A_700 nm_) pH_1.0_ − (A_510 nm_ − A_700 nm_) pH_4.5_] MW* × TV × DF × 1000/(ε × L × SW) (2)

where A corresponds to the absorbance; MW indicates the molecular weight of cyanidin-3-glucoside, equal to 449.2 g/mol^−1^; TV represents the extract total volume; DF is the dilution factor; ε corresponds to the extinction coefficient, which equals 22,400 L/(mol × cm); L is the cuvette length of 1 cm; and SW represents the weight of sample or starting material. The results were determined as mg of cyanidin-3-glucoside equivalent (CGE)/g DE.

#### 2.4.4. Radical Scavenging Activity

The antioxidant capacity was evaluated by employing the 1-diphenyl-2-picrylhydrazyl (DPPH) radical method, with few changes with respect to that previously described [24]. In detail, 0.5 mL of extract solution was added to 4.5 mL of 0.1 mM ethanolic solution of DPPH. The obtained mixture, after being placed in the dark at room temperature for 30 min, was analyzed at 517 nm with the same previously used spectrophotometer. Trolox was employed as a reference compound, and the antioxidant activity was expressed as mg Trolox equivalent (TE)/g DE. 

#### 2.4.5. Vitamin C Content

The content of vitamin C in the rosehip extracts was assessed through the HPLC-DAD method reported by Caprioli et al. [28]. Precisely, 2 mg of DE was dissolved in 1 mL of distilled water, and the samples were filtered through a 0.45 µm PTFE filter prior to HPLC-DAD analysis. The vitamin C quantification was carried out through a Hewlett Packard (Palo Alto, CA, USA) HP-1090 Series II, consisting of an autosampler and a binary solvent pump, with a diode-array detector (DAD). The mobile phases, represented by water with 0.1% formic acid (90%) and methanol with 0.1% formic acid (10%), were used under isocratic conditions, with a flow rate of 0.5 mL/min. The employed analytical column was a Synergi Polar-RP C18 (4.6 mm × 150 mm, 4 µm) from Phenomenex (Torrance, CA, USA), and the injection volume was 10 µL. The run time was 5 min. UV spectra were acquired between 210 and 400 nm, and the 245 nm wavelength was selected and utilized for the quantification. The results were reported as mg vitamin C/g DE.

The developed HPLC/DAD method was validated, and calibration curves of the analyzed ascorbic acid were constructed by injecting standard solutions at seven different concentrations, namely 1, 5, 10, 20, 25, 50, and 70 mg/L. The calibration curve of the analyzed compound showed a correlation coefficient equal to 0.9971. Three replicates for each concentration were performed during 5 days, and the relative standard deviations (RSDs) ranged from 1.2 to 2.4% for run-to-run precision and from 2.8 to 5.4% for day-to-day precision. Limit of detection (LOD) and limit of quantification (LOQ) were estimated on the basis of 3:1 and 10:1 signal-to-noise ratios obtained with standards containing the compounds of interest at low-concentration levels; they were 0.15 and 0.50 mg/L, respectively.

## 3. Results and Discussion

### 3.1. DoE Analysis of MHG Process

**Figure 1:**
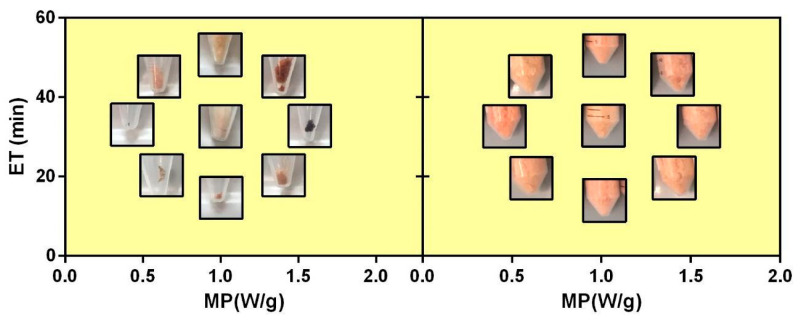
Effect of the MHG parameters microwave power (MP), extraction time (ET), and moistening pretreatment (MO) with (right panel) and without MO (left panel) on the macroscopic aspect of the dry extract (DE).

The rosehips processed using the MHG procedure gave a juice, although after the extraction runs (Table 1) execution appeared immediately evident as the water moistening pretreatment (MO) noticeably affected some specific features of the juice and dry extract. In the absence of this pretreatment, the amount of juice extracted was markedly low, and the quantity of dry extract (DEs) recovered after lyophilization was 2 orders of magnitude lower with respect to the one obtained using the pretreatment. Even visually, the DEs obtained with or without the pretreatment were different; in the presence of the MO, all the DEs had a similar orange color for all the runs (11–20 of Table 1), while, in the absence of the MO, the lyophilized products had different colors ranging from pale yellow up to a very dark red. The darkest colors were observed for the DE recovered from the extraction runs carried out at the longest ET and highest MP (runs 4 and 6). In both these cases, the residual rosehips within the microwave reactor were burned. The colors of all the DEs as a function of the MHG experimental parameters are reported in Figure 1.

**Figure 2:**
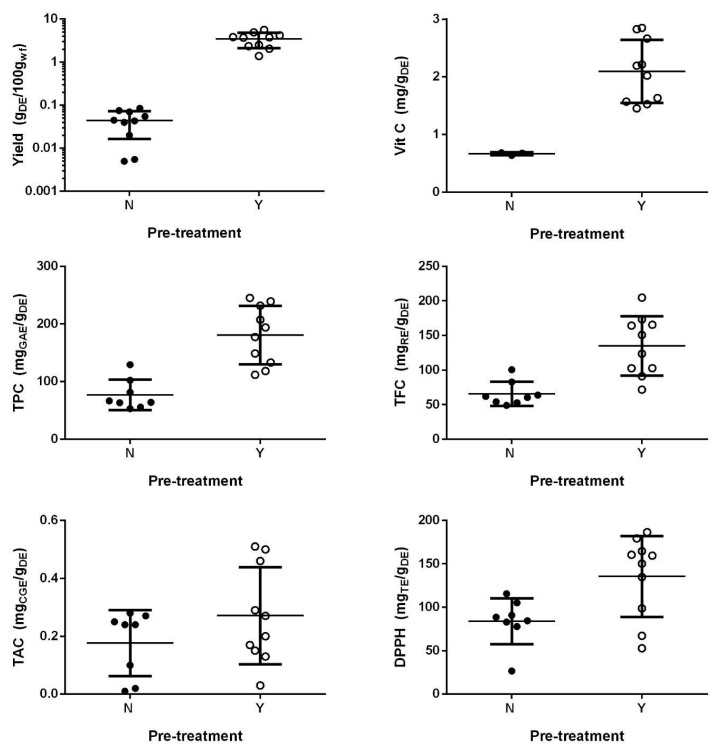
Effect of the moistening pretreatment (MO; N: no pretreatment, Y: pretreatment) on all measured features of the dry extract (extraction yield, vitamin C content, TPC, TFC, TAC, and DPPH). The symbols represent the results of each single run, while the lines are the mean and the standard deviation of all the runs. For sake of clarity, it has been decided to use a logarithmic axis to allow the numerical visualization of the yield values.

In addition, the number of DE components endowed with antioxidant activity was remarkably lower in the extracts obtained without the pretreatment (Figure 2), particularly evident in the case of vitamin C. In some cases, the amount of DE was so low that the execution of the colorimetric tests or the vitamin C quantification was impossible. 

These results do not agree with previous studies where the MHG extraction was effective without any preliminary pretreatment or solvent addition [15,17]. However, the discrepancy is only apparent. In fact, the previous literature results refer to juice extraction from onions, strawberries, and blackberries, all vegetable matrices having a water content higher than 77%, while for the rosehips the water content is remarkably lower (55%). So, considering that the microwaves act on polar molecules like water, it is likely that below a certain water content these technologies become ineffective if a preliminary MO is not carried out. In this situation it is useless to analyze the DoE as planned in Section 2.3.1 since the factor MO is so important that it would hide all the others. Thus, to evaluate how the other factors (MP and ET) affect the MHG process of rosehips, it has been decided to remove all the runs without the MO from the DoE analysis. The new design is constituted by 10 runs (from 11 to 20 in Table 1) and represents a standard CCD for 2 numerical factors (a CCD for 2 numerical factors and a categorical one is the sum of two CCDs for 2 numerical factors each of one set at one of the two levels of the categorical factor). Obviously, the MO is the essential prerequisite for MHG extraction of rosehips. The results of the multilinear regression of the CCD are reported in Table 2.

**Figure 3:**
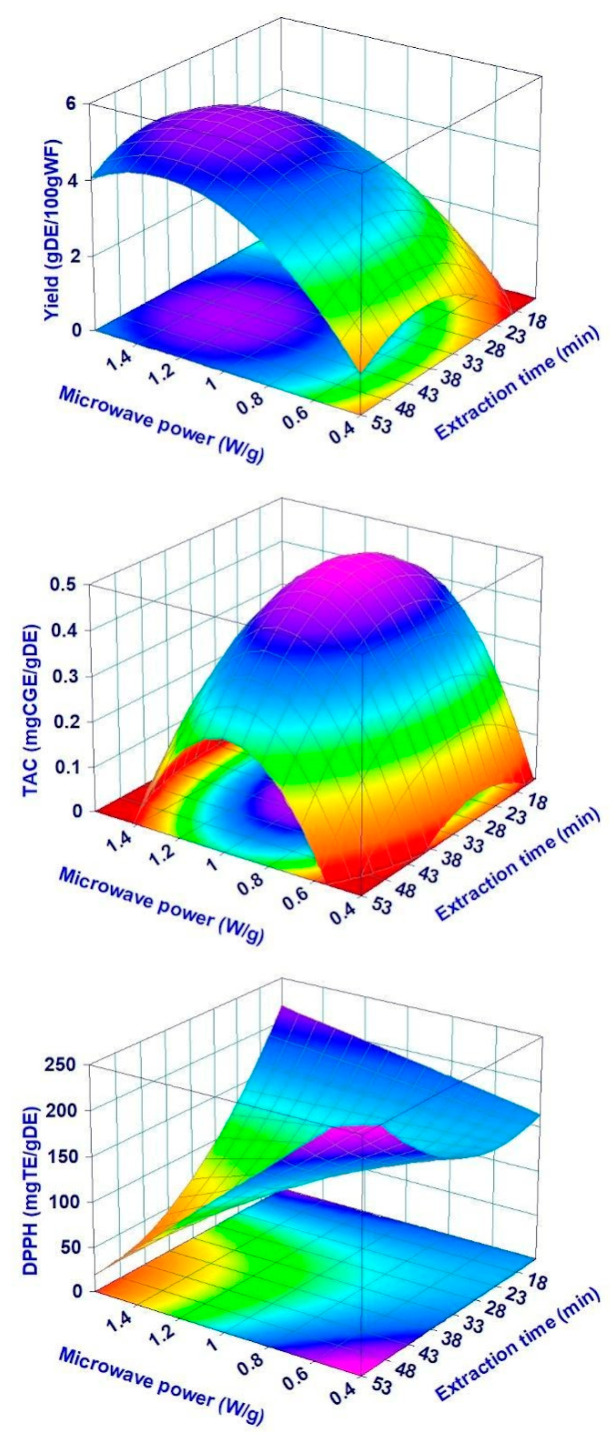
Prediction (surface plot) of the yield, TAC, and DPPH of the dry extracts as a function of MHG parameters microwave power (MP) and extraction time (ET) for samples subjected to the moistening pretreatment.

Among all the responses considered, only the yield, the anthocyanin content, and the radical scavenging activity were described satisfactorily by the full quadratic model or its reduced forms, with values of adjusted R^2^ equal or higher than 0.72, significant regression, and absence of anomalies on the residual analysis (not shown). For all the others considered responses, such as the content of polyphenols, flavonoids, and vitamin C, no statistically significant relationship could be found with the experimental parameters studied. For polyphenols and flavonoids, the data seem to possess a certain random variability, which makes it difficult to define a suitable model. Previous studies carried out in a similar manner on aqueous extracts from MAE showed that the contents of these two classes of compounds are sometimes difficult to be related to the experimental conditions applied [15,18]. On the other hand, the content of vitamin C was almost constant for all the extraction runs. The effect of the experimental parameters on the responses modeled by the full quadratic model is reported in Figure 3 using the response surfaces.

The yield increases as a function of MP and ET up to a plateau at around 1.2–1.4 W/g and 38–48 min, respectively. The surface has a shape similar to that reported for the juice of black mulberry [15] and the aqueous extract of hemp [18], suggesting that the parameters ET and MP during an MHG process act in a similar manner independently of the vegetable matrix considered. Also, for anthocyanins, the surface shape is comparable with that previously reported, with a maximum at low ET and average MP, as a result of their thermal stability [29]. From the other side, the DPPH surface showed different trends compared with the literature ones. Specifically, the DPPH values are similar at all the conditions except for the extreme ones, where they are two- to three-fold lower. The DPPH values depend on the presence of antioxidant molecules, and the response surface suggests that the dry extracts with the highest DPPH values are richer in terms of anthocyanins.

### 3.2. MHG Optimization

**Figure 4:**
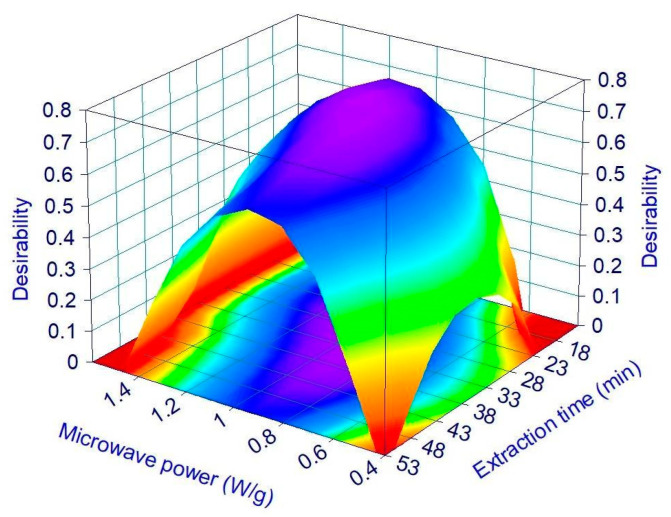
Effect of the MHG parameters on the desirability values.

The MHG process optimization has been performed to maximize all the three modeled responses at the same time. The composite desirability function has been calculated, and its response surface is reported in Figure 4.

As expected from the shape of surfaces reported in Figure 3 for the single responses, the desirability function has a maximum at intermediate values of MP and ET, while it decreases at very low values as one moves away from the center of the experimental domain. The maximum obtainable value of desirability is 0.72, corresponding to a run carried out at a MP of 1.04 W/g for 26.3 min. However, since the central point of CCD (runs 19 and 20 in Table 1) has a desirability practically equal to the maximum obtainable (0.71 vs. 0.72), it has been decided to choose the experimental conditions of the central point as the best ones. To confirm the validity of the model, the central point is repeated for a further two runs (21 and 22), and the average results of all the four replicates were compared with the model prediction. All the measured values fall within the 95% prediction interval determined by the models and even within the 95% confidence interval calculated by the models for the three responses yield, TAC, and DPPH. These results suggest the prediction reliability of the defined models.

**Figure 5:**
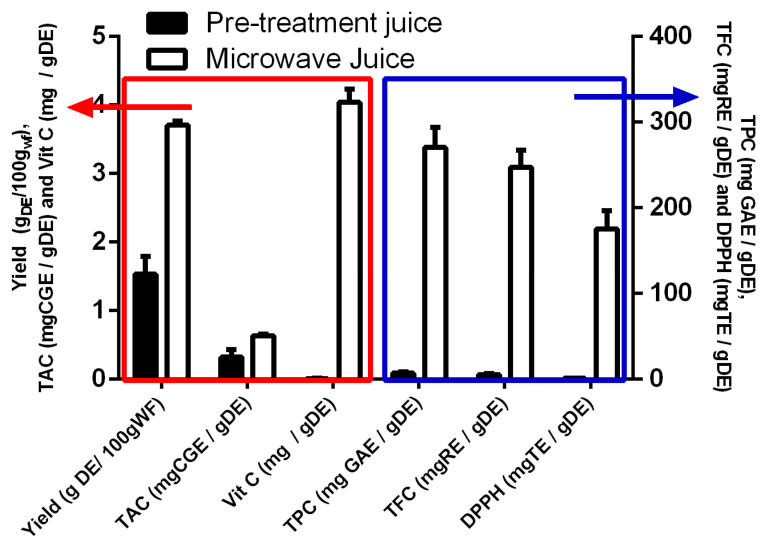
Comparison of the characteristics of the dry extracts obtained from the juice of the pretreatment (black bars) and from the juice extracted by microwaves (white bars).

Finally, a crucial point to be clarified is the effectiveness of the MHG on the juice extraction of rosehips. In fact, the null results of runs 1–11 could suggest that the extraction is carried out mainly during the preliminary moistening pretreatment and that the microwaves contribute only in a marginal way. To verify this aspect, during runs 21 and 22, the juices collected from the preliminary moistening pretreatment were separated from those collected during the successive microwave treatment. Both juices were lyophilized, and the two DEs obtained were analyzed as for all the runs of the DoE. The obtained results clearly indicate that the DE obtained from the juice extracted by microwaves contributes the vast majority to the yield and polyphenol, flavonoid, anthocyanin, and vitamin C contents, as well as to the antioxidant activity (Figure 5).

### 3.3. MHG vs. Conventional Extractions

**Figure 6:**
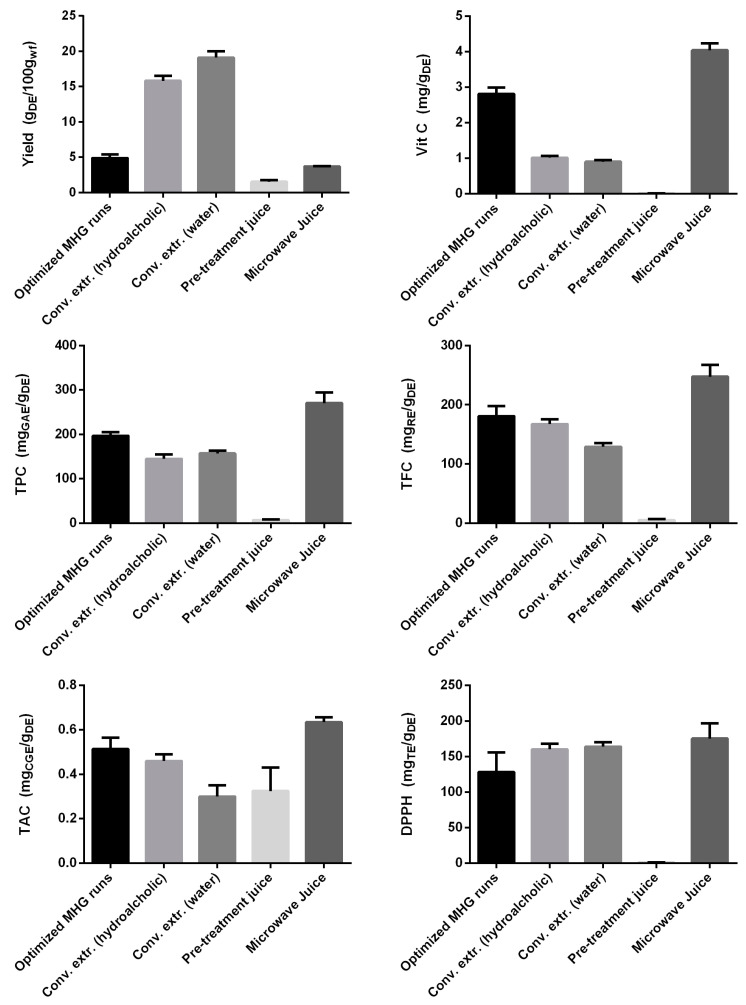
Comparison of the characteristics of the dry extracts obtained from MHG (optimized run, *n* = 4), conventional extraction methods, and from the juice of the pretreatment (*n* = 2) and from the juice extracted by microwaves (*n* = 2).

Rosehip extraction is normally carried out using the maceration procedure in water or hydroalcoholic mixtures [30]. For this reason, two conventional extractions, one in water and the other one in water/ethanol mixture 1:1, were performed, and the DE features were determined. The comparison between the results of the conventional extracts and those of the optimized MHG extraction (runs 19–22) is reported in Figure 6 as well as the results determined for DE obtained separating the juice collected from the preliminary moistening and the proper microwave treatments (runs 21 and 22).

Conventional extractions perform better than MHG in terms of yield, which is 3–4 times higher. However, the MHG process assures a higher concentration of polyphenols, flavonoids, anthocyanins, and vitamin C. Such differences are still more marked if the DE obtained exclusively from the microwave treatments is considered (see microwave juice in Figure 6). These results suggest that conventional extractions are less selective towards hydrosoluble phenolics and vitamin C with respect to MHG. On the other hand, the high yield of these extracts may be given by a higher content of sugars and other low-molecular-weight compounds.

The level of vitamin C in the rosehips used in this study to obtain the optimized MHG extract was lower than that found in several other works regarding conventional extractions from rosehips. In fact, the amount of vitamin C detected in the literature is on average about 0.4% in fresh-weight fruits [6,31,32]. Nevertheless, other reports are present in which the content of vitamin C in rosehips is similar [33] or even lower [30] than that registered in the present study for the optimized MHG extract. Such frequently observed variations are probably due to the different extracted plant material and extraction process, diverse storage and processing conditions, and a certain variability in variety, ecological factors, altitude, and harvest time [30]. Notably, in our work, the vitamin C level in the optimized MHG extract was more than 2 times higher with respect to that obtained in conventional extracts. 

## 4. Conclusions

Rosehips are among the richest natural sources of vitamin C and are widely used in the nutraceutical and food supplement industry. In the present work, MHG extraction was applied and statistically optimized for the first time for rosehips by identifying the best operative conditions in terms of microwave power and extraction time able to assure the highest yield, anthocyanin recovery, and antioxidant activity of the rosehip extracts. The water moistening pretreatment was found to be an essential factor for obtaining the extracts. Notably, the effectiveness of the MHG on the juice extraction of rosehips was demonstrated since the extract obtained from the juice extracted by microwaves contributed the vast majority to the yield and polyphenol, flavonoid, anthocyanin, and vitamin C contents, as well as to the antioxidant activity, with respect to the juice obtained by the moistening pretreatment. In addition, the current study showed for the first time that MHG is a valid alternative to standard extraction methods in order to obtain rosehip extracts rich in vitamin C and phenolic compounds. The levels of these bioactive constituents using this method were higher than those detected with standard extractions, although the latter assured an overall higher yield of extracts. Therefore, MHG may be employed on an industrial level to produce vitamin-C-based food supplements from rosehips.

## Figures and Tables

**Table 1:** Experimental runs performed according to the CCD. Each single factor’s set is reported as both coded and uncoded variables.

Run	Point Type ^a^	Coded Variables ^b^			Uncoded Variables		
		MP (W/g)	ET	MO	MP (W/g)	ET (min)	MO (y or n)
1	F	−1	−1	1	0.6	20	n
2	F	+1	−1	1	1.4	20	n
3	F	−1	+1	1	0.6	45	n
4	F	+1	+1	1	1.4	45	n
5	A	−1.41	0	1	0.4	33	n
6	A	+1.41	0	1	1.6	33	n
7	A	0	−1.41	1	1	15	n
8	A	0	+1.41	1	1	50	n
9	C	0	0	1	1	33	n
10	C	0	0	1	1	33	n
11	F	−1	−1	2	0.6	20	y
12	F	+1	−1	2	1.4	20	y
13	F	−1	+1	2	0.6	45	y
14	F	+1	+1	2	1.4	45	y
15	A	−1.41	0	2	0.4	33	y
16	A	+1.41	0	2	1.6	33	y
17	A	0	−1.41	2	1	15	y
18	A	0	+1.41	2	1	50	y
19	C	0	0	2	1	33	y
20	C	0	0	2	1	33	y

^a^ The “point type” column describes whether a certain set of experimental conditions depicts a factorial (F), axial (A), or central (C) point in the CCD experimental domain. ^b^ The coded variables 1, 1.41, and 0 represent the point type as defined in the “point type” column. The coded variable with value 1.41 corresponds to the α value, that is the radius of a circle inscribing a square having the length sides equal to 1. The abbreviations are as follows: MP, microwave irradiation power; ET, extraction time; MO, water moistening pretreatment.

**Table 2:** Best mathematical models for all the measured responses and their evaluation parameters: coefficients of determination (R^2^_adj_ and R^2^_pred_), Mallows’ Cp statistic, and ANOVA results (*p*-values of regression and lack of fit). The models indicated in this table are only those possessing a statistically significant regression (*p*-value regr < 0.05) and an adjusted multiple regression coefficient higher than 0.5.

Response	Best Model ^a^	R^2^	R^2^_adj_	R^2^_pred_	Mallow’s Cp	*p*-Value Regr ^b^	*p*-Value LOF ^b^
Yield (g_DE_/100g_WF_)	y = −8.59 + 11.92 MP + 0.318 ET − 4.70 MP^2^ − 0.004 ET^2^	0.88	0.79	0.47	4.51	*	ns
TPC (mg_GAE/_g_DE_)	None of the obtained models can describe the relationships between the MHG parameters and TPC						
TFC (mg_RE/_g_DE_)	None of the obtained models can describe the relationships between the MHG parameters and TFC						
TAC (mg_CGE/_g_DE_)	y = −1.045 + 2.458 MP − 0.017 ET − 0.021 W − 1.297 MP^2^ − 0.000384 ET^2^	0.88	0.78	0.50	5.68	*	ns
DPPH (mg_TE/_g_DE_)	y = 162 + 128.9 MP + 2.85 ET + 0.1245 ET^2^ − 6.47 MP * ET	0.80	0.72	0.67	4.78	*	ns
Vit. C (mg/g_DE_)	None of the obtained models can describe the relationships between the MHG parameters and Vit. C content						

^a^ The models are described through the coefficients calculated from the uncoded variables. ^b^ The results of *p*-value columns are indicated as follows: ns = *p* > 0.05; * 0.05 < *p* < 0.01.

## Data Availability

Data are available on request.
